# Finite element graft stress for anteromedial portal, transtibial, and hybrid transtibial femoral drillings under anterior translation and medial rotation: an exploratory study

**DOI:** 10.1038/s41598-024-61061-y

**Published:** 2024-05-24

**Authors:** Roberto Yañez, Rony Silvestre, Matias Roby, Alejandro Neira, Camilo Azar, Samuel Madera, Alejandro Ortiz-Bernardin, Felipe P. Carpes, Carlos De la Fuente

**Affiliations:** 1 Biomechanics unit, Innovation centre, MEDS clinic, Santiago, Chile; 2 Orthopaedic knee service, MEDS clinic, Santiago, Chile; 3 Escuela de Kinesiologia, Facultad de Medicina y Ciencias de la Salud, Universidad Mayor, Santiago, Chile; 4 Ingenieria Civil Mecanica, Facultad de Igenieria, Universidad de Chile, Santiago, Chile; 5 Laboratory of Neuromechanics, Universidade Federal do Pampa, Uruguaiana, Brazil; 6 Exercise and Rehabilitation Sciences Institute, Postgraduate, Faculty of Rehabilitation Sciences, Universidad Andres Bello, Santiago, RM Chile

**Keywords:** Anterior cruciate ligament, Transtibial, Transportal, Surgery, Biomechanics, Reconstruction, Rehabilitation

## Abstract

Stress concentration on the Anterior Cruciate Ligament Reconstruction (ACLr) for femoral drillings is crucial to understanding failures. Therefore, we described the graft stress for transtibial (TT), the anteromedial portal (AM), and hybrid transtibial (HTT) techniques during the anterior tibial translation and medial knee rotation in a finite element model. A healthy participant with a non-medical record of Anterior Cruciate Ligament rupture with regular sports practice underwent finite element analysis. We modeled TT, HTT, AM drillings, and the ACLr as hyperelastic isotropic material. The maximum Von Mises principal stresses and distributions were obtained from anterior tibial translation and medial rotation. During the anterior tibia translation, the HTT, TT, and AM drilling were 31.5 MPa, 34.6 Mpa, and 35.0 MPa, respectively. During the medial knee rotation, the AM, TT, and HTT drilling were 17.3 MPa, 20.3 Mpa, and 21.6 MPa, respectively. The stress was concentrated at the lateral aspect of ACLr,near the femoral tunnel for all techniques independent of the knee movement. Meanwhile, the AM tunnel concentrates the stress at the medial aspect of the ACLr body under medial rotation. The HTT better constrains the anterior tibia translation than AM and TT drillings, while AM does for medial knee rotation.

## Introduction

Around 10–15% of Anterior Cruciate Ligament Reconstruction (ACLr) failures mainly occur by tunnel placement, graft fixation, and graft properties^1–3^. An example is the reduced ultimate strength caused by irradiation on ACLr grafts, compromising the mechanical graft properties. In this sense, the ACLr femoral drilling^4^, which importantly determines the graft orientation^5^, joint constraints, and stress distributions on the graft^5^, would be the most challenging procedure during an ACLr. A more verticalized graft tightens it in the anteroposterior axis (or roll axis), while a more horizontalized graft does for the craniocaudal axis (or yaw axis)^5–7^. Because of that, the femoral tunnel orientation can reduce the rotatory arm, particularly in the transverse plane^8–10^. Consequently, the femoral tunnel orientation might alter the stress distributions and concentration on the ACLr graft, which are relevant factors to explain probable places of ACLr failure (rupture)^11^.

The transtibial (TT) and the anteromedial portal (AM) tunnel are the two typical drillings for ACLr^8^. The TT is a verticalized technique with recognized good long-term outcomes but a higher risk of non-anatomical femoral attachment. At the same time, the AM is a deflected technique at the femoral tunnel with unconstrained anatomic placement^12^. However, the hybrid transtibial tunnel (HTT), which aims to avoid shortened graft and to promote a more centered femoral footprint attachment, has captured attention in the last few years because their coronal (or frontal) angulation is between the angulation of TT and the AM^6,12,13^. The HTT uses a flexible sheathed wire guide inserted through a standard medial portal to permit a more horizontalized attachment than TT^6^. The AM, TT, and HTT techniques have an average sagittal (or lateral) orientation of around 146.3°, 155.4° and 158.8°^13^, and an average coronal (or frontal) orientation of around 100.5°, 129.4° and 111.0°, respectively^13^. Conversely, the AM tunnel would induce a stress concentration and higher forces at the ACLr graft near the femoral tunnel^6^, and the anterior tibial translation and medial knee rotation movements have been established as crucial independent non-contact ACLr rupture causes^14^.

A robust method providing biomechanical insights for surgical designs is the finite element analysis (FEA)^15^. It simplifies the analysis by dividing complex geometric into smaller ones^15^. Thus, the FEA would allow a better understanding of the maximum principal stresses and distributions on the ACLr graft for TT, HTT, and AM femoral tunnels under anterior translation and medial knee rotation. Although the resultant force at the level of femoral deflection angle in the femoral tunnel has been estimated from in vitro testings for knee flexion^6^, we found no information about FEA for TT, HTT, and AM under isolated anterior tibial translation and medial knee rotation. FEA, in this context, would help explore the stress distribution among these surgical designs. Therefore, we aimed to describe the maximum principal stresses and the stress distributions on the graft for TT, AM, and HTT techniques during the anterior tibial translation and medial knee rotation in a finite element model. We hypothesize that comparing the three techniques, i) the TT drilling elicit the lowest peak stress for the anterior tibial translation, and ii) the AM drilling elicit the lowest peak stress for medial knee rotation.

## Material and methods

### Study design

In this in silico study, we reconstructed the knee bones through FEA on a healthy young adult with a non-medical record of ACL rupture and regular sports practice from knee X-ray computed tomography (CT) images. Then, we modeled the TT, AM, and HTT drillings using the same ACLr graft. The knee model was submitted to anterior tibial translation and medial knee rotation, obtaining the maximum Von Mises principal stresses and the location of the stress. This study follows STROBE guidelines and principles^16^ and followed human ethical requirements for human research (see details in the “Ethical declaration”).

### Participant

A healthy knee without ligament rupture from a volunteer of 26 years old, body mass 71 kg, height 1.73 m, and body mass index 23.7 kg m^−2^ was collected in a CT (Brivo CT 385 series, GE Healthcare, USA) using 16-slices with a 0.62 mm resolution and pixel spacing of 0.35 mm × 0.35 mm. The sample size was determined according to the literature sample size for studies using finite element simulations (n = 1)^8,17^. The participant selection was based on age between 20 and 30 years old, non-injured ACL records, generally good health condition assessed by a senior orthopedic medical surgeon (RY), and regular sports practice (twice a week). The exclusion criteria were musculoskeletal acute injury records from the last year, any orthopedic, pathology, metabolic or rheumatic condition, pharmacological use, posterior tibial slope ≥ 10°^18^, no ligament laxity, no genu-recurvatum^19^, positive Lachman, anterior drawer, or pivot shift test, increased navicular drop^19^, decreased intercondylar notch width and volume^20^, increased meniscal slope^19^, discoid meniscus, increased knee abduction angle and intersegmental moment during jumping^19^, increased ground reaction force during jumping^19^, increased trunk displacement during jump landing^19^, increased knee hyperextension^19^, quadriceps force asymmetry > 12%, increase in side-to-side differences^19^, and increased body mass index^19^. The criteria were measured by a senior biomechanist (RS), radiologist (NG), and orthopedic medical surgeon (RY).

### Knee joint models

**Figure 1:**
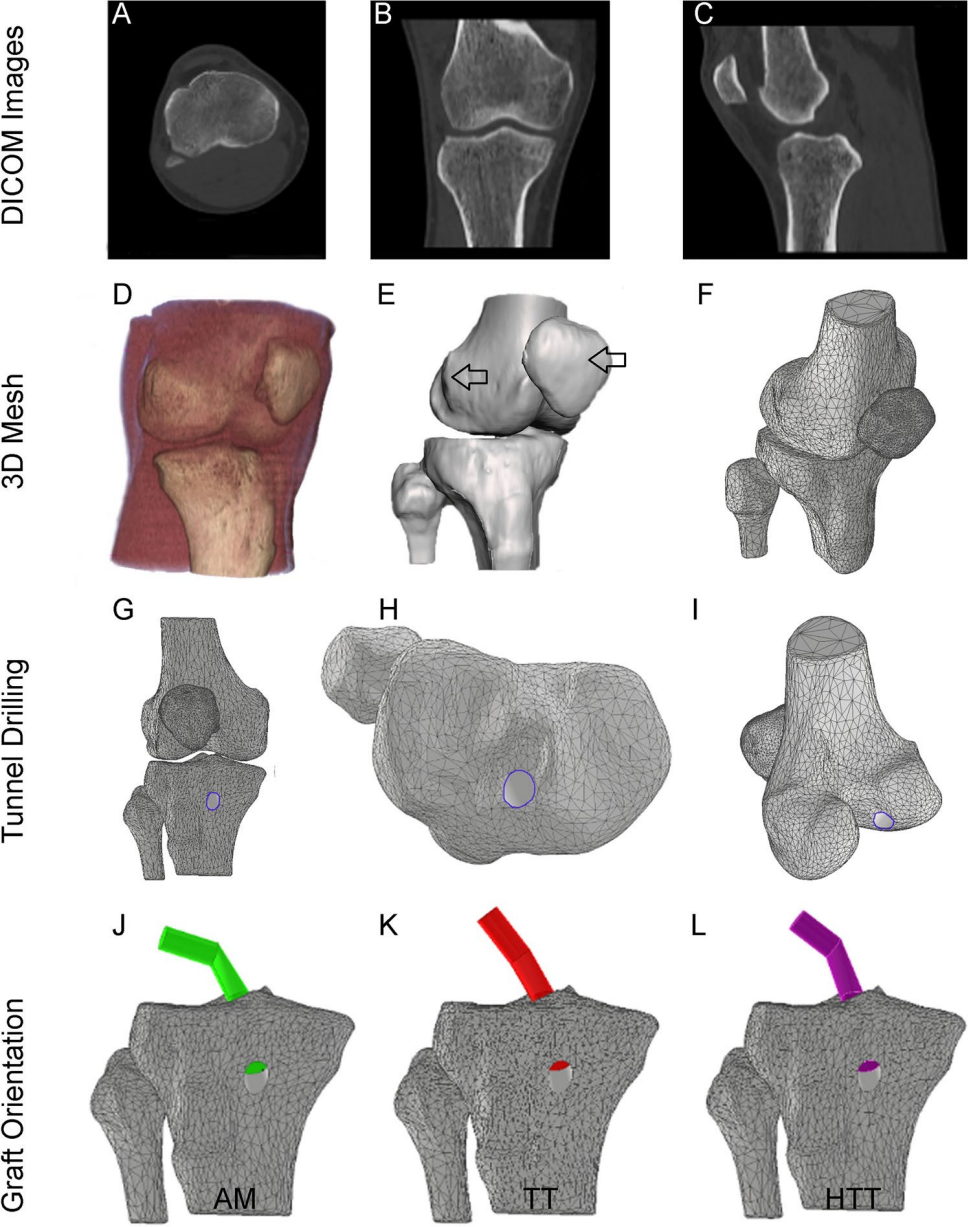
Knee Modeling procedures. (**A**–**C**) DICOM Images obtained from the participant. (**D**) Whole reconstructed knee of the participant. (**E**) First STL of the knee model. (**F**) Optimized STL model with independent bones (no attached between them). (**G**–**I**) Tunnel drilling simulation in different planes. (**J**) Anteromedial portal (AM) graft orientation. (**K**) Transtibial (TT) graft orientation. (**L**) Hybrid transtibial (HTT) graft orientation. The arrows show the application point of the force that caused anterior tibial translation and the medial knee.

The DICOM CT data (Fig. 1A–C) were segmented as bone by threshold intensity (Fig. 1D) to create a 6 Degree of Freedom (DoF) ACLr graft rigidly attached to the femur and tibia (Fig. 1E and F). Each bone was manually selected, and a pixel-grown region was applied during the segmentation. The next step was smooth filtering (joint smoothing) through the Slicer 3D Slicer software version 5.0.0 (The Brigham and Women’s Hospital, Inc., USA). For a complete bone separation between the fibula, tibia, femur, and patella, the obtained 3D bone model was manually edited using Mesh-mixer software version 3.5 (Autodesk, USA) to eliminate internal residuals until independent surface solids (only cortical bones) were obtained (Fig. 1F). The model only considered the ACLr graft and bone elements in agreement with previous reports^17^ to explore the direct effects of anterior tibial translation and media knee rotation.

### Femoral and tibial tunnels

The tibial and femoral drilling was based on Troffa et al.^13^ using boolean operations. A circular section with a 9 mm diameter was made. The first drilling was made in the tibia (Fig. 1G and H). Then, the subsequent drilling was made in the femur (Fig. 1I) with a sagittal (or lateral) orientation of 146.3°, 155.4°, and 158.8° for AM portal, TT, and HTT, respectively^13^, while the coronal orientation (or frontal) was 100.5°, 129.4°, and 111.0° for AM portal, TT, and HTT, respectively^13^ (Fig. 1J–L). Finally, an ACLr graft with a 9 mm diameter was attached with a width of 25 mm (Fig. 1) based on Achilles graft preparation^21^. The node and total elements were 8812/42,454, 11,482/53,070, and 9776/48,076 units for the AM portal, TT, and HTT, respectively. We iterate the mesh size until convergence for stable stress and displacement, obtaining the previously described nodes and elements. All surgical models on the finite element were made using Inventor software version 26.0 (Autodesk, USA).

### Materials properties

The tibial and femoral properties were a rigid body material with a Young module of 0.4 GPa and a Poisson coefficient of 0.33^22^. The ACLr was modeled as isotropic hyperelastic (no linear) material with Veronda-Westmann coefficients of α = 0.3 Mpa and β = 12.20^22^. The femoral and tibial attachments were set as rigid contacts.

### Boundary conditions

A knee model underwent independent anterior translation and medial rotation, allowing for the determination of maximum Von Mises principal stresses and the corresponding stress locations. The boundary conditions were 1 degree of freedom (DoF) in the anteroposterior axis (or roll axis) of the femur, 1 DoF in the craniocaudal axis (or yaw axis) of the femur, and 6 DoF (3 translations and three rotations) for ACLr. After that, a force of 120 N was applied at the center of inertia from the anterior aspect of the femur with a posterior direction on the femur, causing an anteriorization of the tibia (Fig. 1E). The load magnitude was close to Cheng et al.^22^, who used an anterior tibial translation that occurs during gait (0.15 times body weight)^22^. Finally, a force of 16 N towards the posterior direction was applied in the most lateral aspect of the lateral femoral condyle, causing an internal rotation moment of 0.8 Nm to test the model (Fig. 1E). We applied these forces empirically until movement was achieved in the physiological movement ranges. The translation force was defined in coherence with previous reports, and the medial moment was 0.4 times compared to previous reports due to our model excluding other secondary stabilizers of the knee^23^. Also, in this study, we did not consider prestrain behavior^24^. All biomechanical simulations were performed using the FEBio software version 3.5.1 (University of Utah & Columbia University, USA).

### Model validation

The fifth case of the Cheng et al.^22^ simulations was used to compare our model. We described the Von Mises stress and deformation distributions for the reconstructed ACLr. Our knee FEA model was studied for the Von Mises stress and deformation distributions for ACLr with 45° in the coronal plane and 60° in the sagittal plane using the AM portal drilling for validation. We chose this because it provided the lowest peak stress and deformation on the bone tunnels and anterior cruciate ligament graft^22^. The graft properties were hyperelastic isotropic material with Veronda-Westmann α = 0.3 MPa, β = 12.20. The boundary conditions were set under the main plane of motion (anterior translation) without considering the medial and valgus moment of Cheng et al.^22^. Because of that, our pattern has limitations, and we recommend caution in extrapolating our results. The Von Mises stress and distributions were expected to be obtained at the lateral aspect of the femoral graft tunnel^22^. All biomechanical simulations were performed using the FEBio software version 3.5.1 (University of Utah & Columbia University, USA).

### Data analysis

The maximum principal stresses and the stress distributions on the ACLr graft were described through descriptive statistics for the anterior tibial translation and medial knee rotation models for TT, AM portal, and HTT techniques. Nephogram maps were scaled to the maximum principal stress of each simulation.

### Ethics declarations

This study was approved by the institutional review board of MEDS clinic (Santiago, Chile). This study was in accordance with Helsinki principles. Signed informed consent was obtained from the participant.

## Results

**Figure 2:**
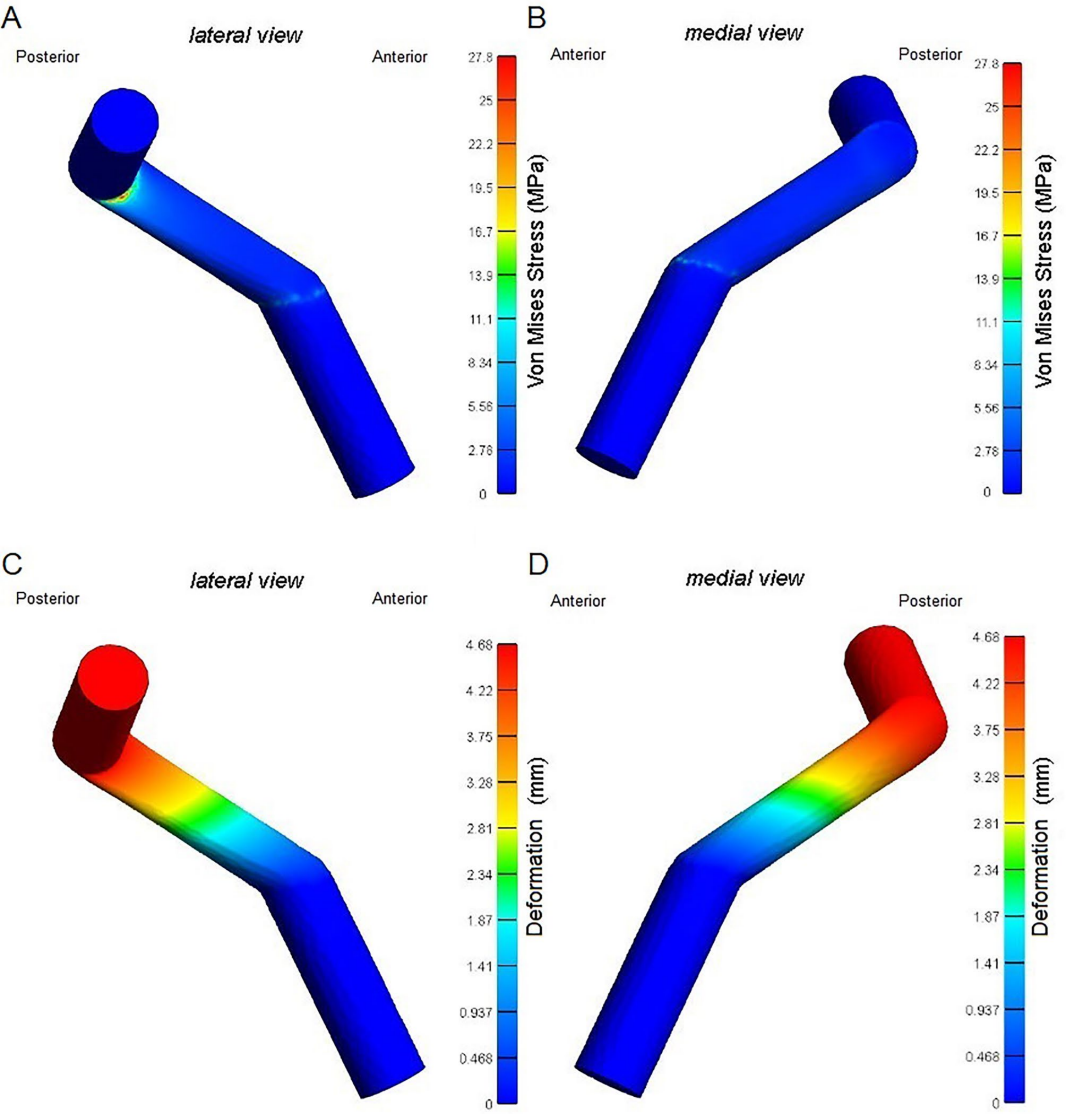
Knee modeling validation outcomes. The figure shows the Von Mises Stress and deformation after 104 N (15% of body weight) applied from the anterior aspect of the femur towards the posterior direction, according to Cheng et al.^22^. The results show the Von Mises stress (**A** lateral view and **B** medial view) and deformation distributions (**C** lateral view and **D** medial view) for Anterior Cruciate Ligament reconstruction for 45° in the coronal plane and 60° in the sagittal plane using the AM portal drilling considering only the anterior translation of the tibia.

Similar to the findings from Cheng et al.^22^ model, in our model, the stress distributions (Fig. 2A and B) were located at the lateral aspect of the ACLr near the femoral tunnel graft attachment. The deformation distributions of our model occurred mainly at femoral tunnel graft attachment (Fig. 2C and D). The maximum principal stress of our model was 27.8 MPa, while Cheng et al.^22^ reported 28.76 MPa.

**Figure 3:**
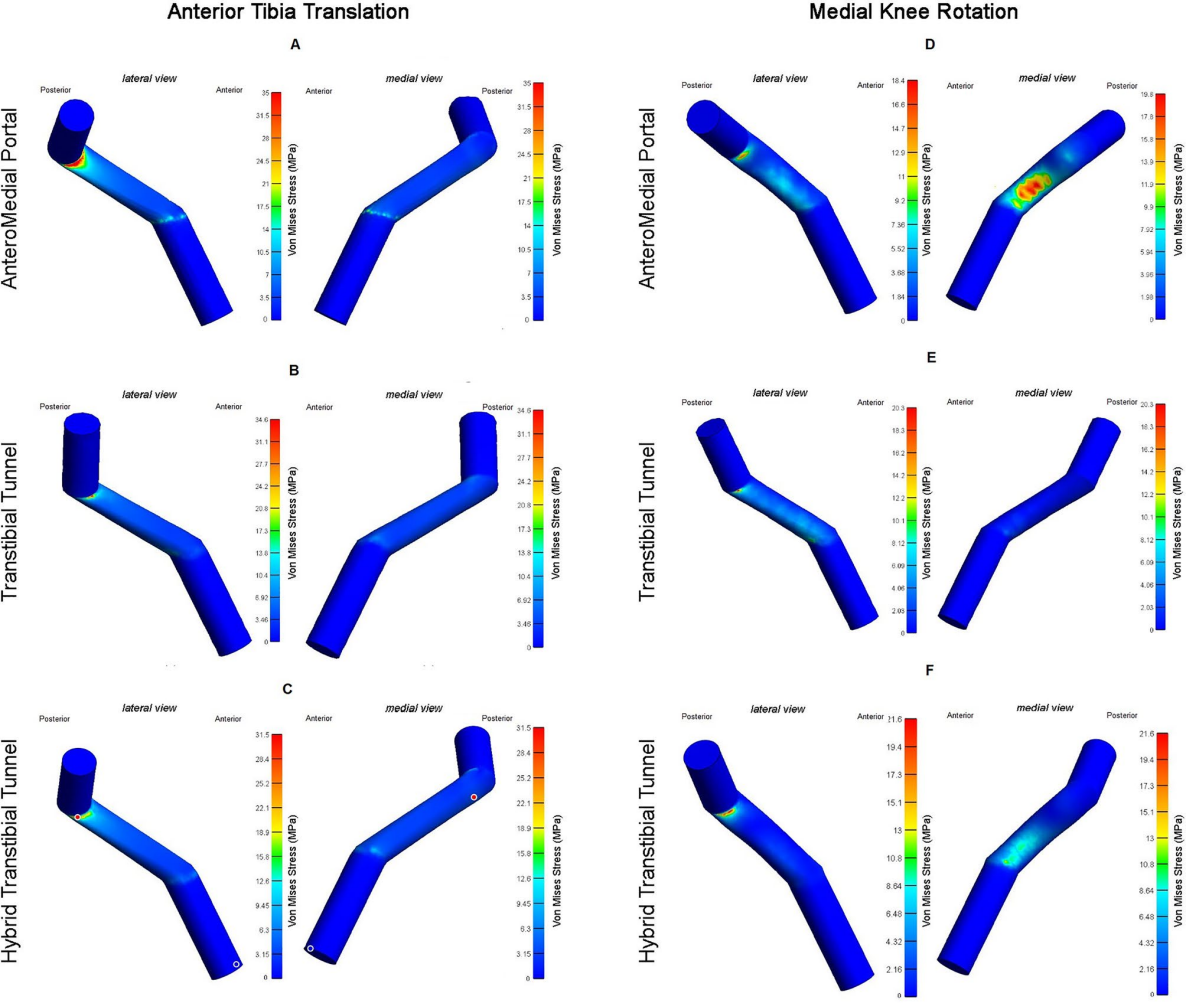
Study outcome for Von Mises Stress for two movements and three techniques. For anterior tibial translation in (**A**) lateral and medial view of the Anteromedial Portal, in (**B**) lateral and medial view of the Transtibial tunnel, in (**C**) lateral and medial view of the Hybrid Transtibial Tunnel. For medial knee rotation in (**D**) lateral and medial view of the Anteromedial Portal, in (**E**) lateral and medial view of the Transtibial tunnel, in (**F**) lateral and medial view of the Hybrid Transtibial Tunnel. The red point is the projection of the maximal stress for HTT technique.

The stress distributions for anterior tibia translation in the AM drilling were concentrated at the lateral aspect of the ACLr graft near the femoral graft attachment (Fig. 3A). The maximum principal stress was 35 MPa. The stress distributions for anterior tibia translation in the TT drilling were concentrated at the lateral aspect of the ACLr graft near the femoral tunnel graft attachment (Fig. 3C). The maximum principal stress was 34.6 MPa. The stress distributions for anterior tibia translation in the HTT drilling were concentrated at the lateral aspect of the ACLr graft near the femoral tunnel graft attachment with the tendency of posterior displacement (red dot in Fig. 3E). The maximum principal stress was 31.5 MPa.

The stress distributions for medial rotation in the AM drilling were concentrated at the lateral aspect of the ACLr graft near the femoral and the medial aspect of the body graft. The maximum principal stress was 17.3 MPa at the medial aspect of the body graft (Fig. 3B). The stress distributions for medial rotation in the TT drilling were concentrated at the lateral aspect of the ACLr graft near the femoral tunnel graft attachment. The maximum principal stress was 20.3 MPa (Fig. 3D). The stress distributions for medial rotation in the HTT drilling were concentrated at the lateral aspect of the ACLr graft near the femoral tunnel graft attachment and at the medial aspect of the ACLr graft near the tibial tunnel attachment. The maximum principal stress was 21.6 MPa at the lateral aspect of the ACLr graft near the (Fig. 3F).

## Discussion

Under mechanical and geometrical controlled conditions, our main findings were that the i) HTT dissipates the anterior tibial translation better; under equal load conditions, the HTT elicits the lowest peak stress compared to the other techniques. Meanwhile, the AM drilling dissipates the medial rotation better; the AM drilling results in the lowest peak stress under equal load conditions compared to the other techniques. During the anterior tibia translation, the stress concentrates mainly on the femoral tunnel graft attachment, with increased peak stress for the AM drilling. ii) During the medial rotation in the AM drilling, the stress concentrates on the femoral tunnel graft attachment and across the lateral graft mid-substance portion. In contrast, the TT and HTT reduce the stress concentration at the lateral graft mid-substance portion during the medial rotation. Therefore, we argue that the stress concentration observed here for ACLr depends on the surgical design (femoral tunnel angulation). However, although the HTT is a surgical alternative to constrain anterior tibial translation, it was not for medial rotation as single-bundle reconstruction. On the other hand, AM constraints the medial rotation movement better without high-stress concentration at the mid-substance of the graft compared with TT and HTT drillings. However, it was not an efficient stress dissipator for the anterior translation of the tibia. Considering that the native ACL provided 82–90% of the total anterior restraint between 0° and 90° knee flexion^25^, the HTT might be an appropriate mechanical alternative to constrain anterior translations, the higher physiological loads experienced by the knee^25–29^. Our comparisons might indicate that techniques like HTT would be designed with an extraarticular lateral tenodesis to better constrain the medial knee rotation^29^. Future FEA studies should explore this surgical alternative.

The overlayed stress distributions between the AM, TT, and HTT drilling partially agree with the stress concentration of other graft models and native ACL previously reported^30^. Previous reports have confirmed the femoral tunnel as a critical stress concentration region^6,17,31^. All our models, independent of the movement, develop a stress concentration at the lateral aspect of the ACLr near the femoral tunnel, and the highest stress was found for the AM technique under the anterior translation of the tibia. Consequently, the attachment graft zone might be the weakest and most critical place for the three drillings, especially during the early postoperative period when tissues had lower resistance and stiffness. Also, our findings would support the slipping of the graft from the attachment^30,32^ caused by early femoral tunnel enlargement. Future research should evaluate the critical role of different attaching techniques and osteosynthesis elements of fixation at femoral tunnels.

The increased mid-substance stress for AM drilling during medial rotation could be explained by the fact that cyclic testing measured greater peak contact pressure when using AM drilling than the TT technique at the tibial tunnel^33^. This AM pattern would respond to a more deflected ACLr graft, tightening more medial fibers of the graft because the medial condyle displaces posteriorly. In contrast, the medial femoral condyle displaces anteriorly during the medial knee rotation. A similar pattern can be observed in previous reports^34^. Both femoral and tibial tunnels create graft-bone contact^8^, putting the graft under higher loads^33^. This pattern has been indicated as a possible graft failure mechanism because it increases the peak contact pressure and should be considered between the ACLr choices^33^, particularly when early rehabilitation is involved. In contrast, the HTT has demonstrated lower loads at tunnels, suggesting an acceptable combination of TT and AM drillings^13^. Also, it is relevant to consider that the ACLr graft gets better and more satisfactory clinical and functional outcomes associated with a lower failure risk when the femoral tunnel is placed more eccentrically in the footprint^35^.

We acknowledge that our research is not without limitations. We identify that the main limitation would be the native geometry of our ACLr graft. However, we have used a similar graft among the techniques based on the recommendations for using the Achilles allograft^21^. Our model validation did not have the same boundary conditions for coronal and transverse knee planes, and it has been considered exclusively the sagittal plane where the main knee motion occurs. The under consideration of these characteristics can explain part of the different values and stress distributions obtained in the validation results. The simplification of the model to an isotropic material and the lack of mesh convergence details and prestrain could also be considered part of the limitation of this study. On the other hand, the biology variability was controlled by simplifying the modeling to a healthy participant and validating our model. Our sample size is commonly used in finite element simulations^8^. Here, it is important to understand that other factors increase graft stress, like the posterior slope^32^, the graft diameter^36^, or graft stiffness, and shape^30^, to mention some of them. In our study, these variables were excluded to better comprehend the effects of surgical design on ACLr stress. Lastly, the modeling attempts to reflect the structures immediately post-surgery, which is more related to early than long-term failure.

In conclusion, HTT drilling suggests better constraining the anterior tibia translation forces, while AM is the most effective drilling to constrain medial knee rotation. Our in silico study permits exploring the advantages of the HTT technique as a surgical alternative for ACLr, but future studies are needed.

## Data Availability

FeBio models are available in the ResearchGate repository of the corresponding author (Carlos De la Fuente, https://www.researchgate.net/profile/Carlos-De-La-Fuente-3).
